# Novel ATPase Cu^2+^ Transporting Beta Polypeptide Mutations in Chinese Families with Wilson's Disease

**DOI:** 10.1371/journal.pone.0066526

**Published:** 2013-07-02

**Authors:** Shaojuan Gu, Huarong Yang, Yong Qi, Xiong Deng, Le Zhang, Yi Guo, Qing Huang, Jing Li, Xiaoliu Shi, Zhi Song, Hao Deng

**Affiliations:** 1 Center for Experimental Medicine, the Third Xiangya Hospital, Central South University, Changsha, China; 2 Department of Neurology, the Third Xiangya Hospital, Central South University, Changsha, China; 3 Department of Neurology, Xiangya Hospital, Central South University, Changsha, China; 4 Department of Physiology, Xiangya Medical School, Central South University, Changsha, China; 5 Department of Gastroenterology, the Second Xiangya Hospital, Central South University, Changsha, China; University of Navarra School of Medicine and Center for Applied Medical Research (CIMA), Spain

## Abstract

Wilson's disease (WD) is an autosomal recessive inherited disorder caused by mutations in the ATPase Cu^2+^ transporting beta polypeptide gene (*ATP7B*). The detailed metabolism of copper-induced pathology in WD is still unknown. Gene mutations as well as the possible pathways involved in the *ATP7B* deficiency were documented. The *ATP7B* gene was analyzed for mutations in 18 Chinese Han families with WD by direct sequencing. Cell viability and apoptosis analysis of *ATP7B* small interfering RNA (siRNA)-treated human liver carcinoma (HepG2) cells were measured by 3-[4,5-dimethylthiazol-2-yl]-2,5-diphenyltetrazolium bromide (MTT) assay and Hoechst 33342 staining. Finally, the expression of B-cell CLL/lymphoma 2 (BCL2), BCL2-associated X protein (BAX), sterol regulatory element binding protein 1 (SREBP1), and minichromosome maintenance protein 7 (MCM7) of *ATP7B* siRNA-treated cells were tested by real-time polymerase chain reaction (real-time PCR) and Western blot analysis. Twenty different mutations including four novel mutations (p.Val145Phe, p.Glu388X, p.Thr498Ser and p.Gly837X) in the *ATP7B* gene were identified in our families. Haplotype analysis revealed that founder effects for four mutations (p.Arg778Leu, p.Pro992Leu, p.Ile1148Thr and p.Ala1295Val) existed in these families. Transfection of HepG2 cells with *ATP7B* siRNA resulted in decreased mRNA expression by 86.3%, 93.1% and 90.8%, and decreased protein levels by 58.5%, 85.5% and 82.1% at 24, 48 and 72 hours, respectively (All *P*<0.01). *In vitro* study revealed that the apoptotic, cell cycle and lipid metabolism pathway may be involved in the mechanism of WD. Our results revealed that the genetic cause of 18 Chinese families with WD and *ATP7B* deficiency-induce apoptosis may result from imbalance in cell cycle and lipid metabolism pathway.

## Introduction

Wilson's disease (WD) is an autosomal recessive disorder of copper metabolism. The disease is caused by mutations in the ATPase Cu^2+^ transporting beta polypeptide gene (*ATP7B*), a cellular copper transporting ATPase. The incidence of WD among different populations varies from 1/30,000 to 1/100,000 [1]. The hallmarks of the disease are the presence of liver disease, neurologic symptoms and Kayser-Fleischer (K-F) rings. The deficiency of *ATP7B* disrupts copper homeostasis, particularly in the liver, by greatly decreasing the ability of exporting excess copper from hepatocytes to bile. Copper accumulation causes severe morphological and functional changes, including cirrhosis, hepatitis and liver failure. There is wide variability in clinical manifestation and age at the onset (from 3 to 70 years) of this disease, and typical biochemical features may not always be present. Therefore, genetic analysis provides the potential for more reliable early diagnosis, and prompt treatment [2]. Genetic analysis reveals at least 506 distinct mutations, including missense and nonsense mutations, deletions and insertions (http://www.wilsondisease.med.ualberta.ca/database.asp), but a detailed mechanistic understanding of copper-induced pathology in WD is still lacking. Knowledge of the distribution of particular mutations may help to design shortcuts for genetic screening strategies of WD. To evaluate the frequency of the *ATP7B* mutations in Chinese Han patients with WD, to explore genotype-phenotype correlations and to possibly unveil the pathways involved in the *ATP7B* deficiency, we screened 18 families with WD and inhibited the *ATP7B* gene expression in human liver carcinoma (HepG2) cells.

## Materials and Methods

### Subjects

**Figure 1:**
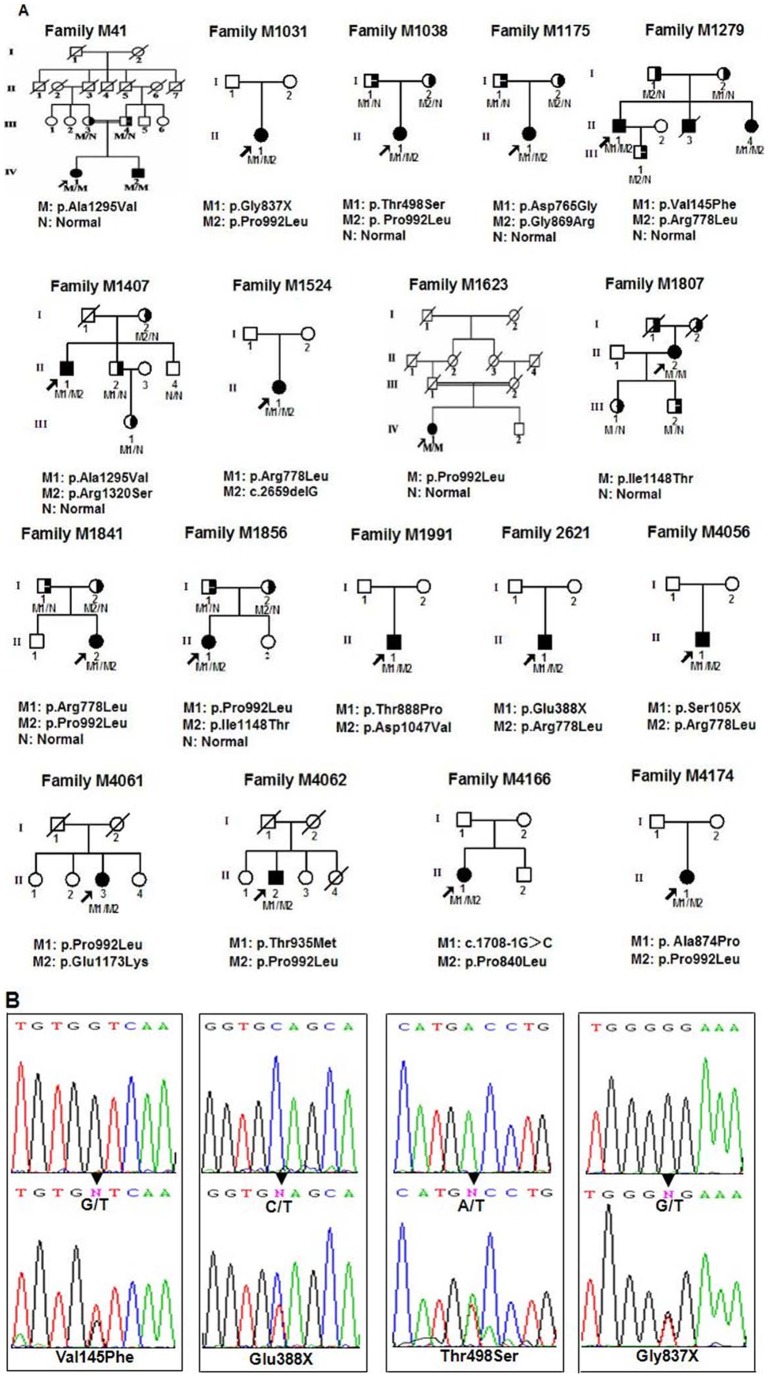
Pedigree figures of WD families (A) and (B) the sequences of the four novel mutations. Arrowheads indicate nucleotide changes.

Eighteen Chinese Han WD families (Figure 1), consisting of 38 family members, and 100 normal age- and ethnic-matched unrelated controls (50 males and 50 females) were enrolled in this study. The mean age of disease onset of patients was 17±10 years (range 2–41 years) (Table 1). All patients were examined and diagnosed at the Third Xiangya Hospital. Their evaluations consisted of medical history, physical examination, ophthalmologic slit-lamp examination, abdominal ultrasound, live function tests, serum copper and ceruloplasmin, and 24-hour urinary copper levels. The Third Xiangya Hospital Institutional Review Board approved this proposal: Identification of the Gene Mutation of Wilson Disease, following the Declaration of Helsinki. Informed consents were written by all participating individuals or guardians on the behalf of the minors/children participants involved in the study.

**Table 1:** Clinical data from the 20 patients with the *ATP7B* gene mutations.

Family	Case	Sex	Age (Y)	Onset age(Y)	Mutations	Symptoms at onset	Extrapyramidal signs	Hepatomegaly	Splenomegaly	Jaundice	K-F ring	SerumCP(mg/L)	Urinary Cu (μg/24 h)
M41	IV:1	F	11	10	Ala1295Val/Ala1295Val	Hepatic	Present	Present	Present	Present	Present	47.2	401
	IV:2	M	10	9	Ala1295Val/Ala1295Val	Hepatic	Present	Present	Present	Present	Present	51.6	346
M1031	II:1	F	18	2	Gly837X/Pro992Leu	Hepatic	Present	Present	Present	Absent	Present	93.7	224
M1038	II:1	F	20	12	Thr498Ser/Pro992Leu	Neurologic	Present	Absent	Absent	Absent	Present	51.7	145
M1175	II:1	F	17	17	Asp765Gly/Gly869Arg	Neurologic	Present	Present	Present	Present	Present	28.9	263
M1279	II:1	M	46	6	Val145Phe/Arg778Leu	Hepatic	Absent	Present	Present	Present	Present	74.2	278
	II:4	F	38	7	Val145Phe/Arg778Leu	Hepatic	Absent	Present	Absent	Present	Present	53.3	194
M1407	II:1	M	44	30	Ala1295Val/Arg1320Ser	Hepatic	Absent	Present	Present	Present	Present	51.7	385
M1524	II:1	F	18	14	Arg778Leu/c.2659delG	Neurologic	Absent	Present	Present	Present	Present	67.5	206
M1623	IV:1	F	36	18	Pro992Leu/Pro992Leu	Hepatic	Present	Present	Present	Absent	Absent	142.9	183
M1807	II:2	F	43	41	Ile1148Thr/Ile1148Thr	Neurologic	Present	Present	Present	Present	Present	72.1	178
M1841	II:2	F	22	22	Arg778Leu/Pro992Leu	Neurologic	Present	Present	Present	Absent	Present	27.4	265
M1856	II:1	F	22	18	Pro992Leu/Ile1148Thr	Neurologic	Present	Present	Present	Absent	Present	52.3	123
M1991	II:1	M	40	40	Thr888Pro/Asp1047Val	Hepatic	Absent	Present	Present	Absent	Present	90	279
M2621	II:1	M	22	20	Gln388X/Arg778Leu	Hepatic	Absent	Present	Present	Absent	Present	38.4	327
M4056	II:1	M	25	11	Ser105X/Arg778Leu	Hepatic	Absent	Present	Present	Present	Present	32.1	290
M4061	II:3	F	21	15	Pro992Leu/Glu1173Lys	Neurologic	Present	Present	Present	Absent	Present	45.2	243
M4062	II:2	M	20	19	Thr935Met/Pro992Leu	Neurologic	Present	Present	Absent	Absent	Present	114.3	189
M4166	II:1	F	20	17	c.1708-1G>C/Pro840Leu	Hepatic	Absent	Present	Present	Present	Present	89.8	378
M4174	II:1	F	14	9	Ala874Pro/Pro992Leu	Neurologic	Present	Present	Present	Absent	Present	70.2	237

Y, years; K-F, Kayser-Fleisher; CP, ceruloplasmin.

### Genetic analysis

Polymerase chain reaction (PCR) amplified all coding regions and intron/exon boundaries of the *ATP7B* gene. The primers sequences are available on demand (RefSeq NG_008806). PCR products were directly sequenced on 3130 Genetic Analyzer. Given that WD is an autosomal recessive disorder with an estimated carrier frequency of 1/90 [1], and the variant found in a patient was considered as a polymorphism other than as a mutation if it exists as a homozygous statue in normal controls.

Haplotype analysis was conducted in families harbored the same mutation with single nucleotide polymorphisms (SNPs) including rs1801243, rs1801244, rs1061472, rs732774, rs1801249, rs2282057 and rs9535795 by sequencing.

### Cell culture, small interfering RNA (siRNA) transfection and RNA extraction

HepG2 cells (ATCC HB-8065, VA, USA) were cultured in Dulbecco's modified Eagle's medium (DMEM, Gibco, Grand Island, NY) supplemented with 10% heat-inactivated fetal calf serum, 1% L-glutamine and 1% penicillin/streptomycin. All experiments were repeated at least three times. *In vitro* transient transfection was done as described previously [3]. *ATP7B* siRNA were purchased from Qiagen. The target sequence was 5′-CCAATTGATATTGAGCGGTTA-3′. Briefly, Cells from passages 10 to 20 were used and they were seeded at 60% confluency into six-well or 96-well dish. The *ATP7B* siRNA and 5.0 μl HiPerFect Reagent (Qiagen, Melbourne, Australia) were diluted into a final volume of 100 μl in Opti-MEM (Gibco, Grand Island, NY), respectively, and gently mixed and incubated at room temperature for 10 min, then 800 μl Opti-MEM was added to the mixture. The above transfection solution was overlaid onto cells at a final concentration of 5 nM siRNA. Transfection of HepG2 cells with AllStars Negative Control siRNA (Qiagen, Melbourne, Australia) served as a negative control. After 12-hour incubation at 37°C in the presence of 5% CO_2_, 2 ml of complete medium with 10% FBS was added to each well of transfected cells to replace transfection solution.

### Real-time polymerase chain reaction (Real-time PCR)

Total RNAs were prepared and cDNA was synthesized by using the SuperScript First-Strand kit (Invitrogen, Carlsbad, CA, USA) as per the manufacturer's instructions. The cDNA samples were amplified by the following primers (Table S1). PCR was done with 1 μg of cDNA and 100 ng/μL of sense and antisense primers in a total volume of 20 μL.

### Western Blot

Cells grown to 90% confluence were harvested and lysed in lysis buffer. Protein (30–50 μg) was heated at 95°C for 5 min, separated by SDS-PAGE and electrophoretically transferred onto PVDF membranes. The blots were blocked for 1 h in 5% milk in TBST and incubated at 4°C overnight with rabbit anti-*ATP7B* polyclonal antibody (Novus Biologicals, Littleton, CO, USA), rabbit anti- minichromosome maintenance protein 7 (MCM7) monoclonal antibody (Millipore Biologicals, Billerica, MA, USA), rabbit anti- sterol regulatory element binding protein 1 (SREBP1) polyclonal antibody (Santa Cruz Biotechnology Inc, Santa Cruz, CA, USA), mouse anti- B-cell CLL/lymphoma 2 (BCL2) polyclonal antibody (Santa Cruz Biotechnology Inc, Santa Cruz, CA, USA), mouse anti- BCL2-associated X protein (BAX) polyclonal antibody (Santa Cruz Biotechnology Inc, Santa Cruz, CA, USA) and mouse anti-β-actin polyclonal antibody (Santa Cruz Biotechnology Inc, Santa Cruz, CA, USA). The membrane was washed three times with PBS and then incubated for 1 h with 1000-fold diluted horseradish peroxidase-conjugated anti-rabbit IgG (Amersham, Little Chalfont, Buckinghamshire, England) to detect *ATP7B*, MCM7 and SREBP1, and horseradish peroxidase-conjugated anti-mouse IgG (Amersham, Little Chalfont, Buckinghamshire, England) to detect BCL2, BAX and β-actin, respectively. Immunoreactive proteins were visualized using enhanced chemiluminescence (Millipore Biologicals, Billerica, MA, USA).

### Analysis of cell viability

HepG2 cells were seeded per well in a 96-well plate and incubated (37°C, 5% CO_2_) for 12 h, then transfected with 5 nM *ATP7B* siRNA for 24 h and recovery for 3 h with cell culture medium. Copper sulfate (0, 50 and 200 μM; Sigma, St Louis, MO, USA) at various concentration was added for 24 hours. The supernatant was aspirated and 200 μl medium mixed with 3 μl 3-[4,5-dimethylthiazol-2-yl]-2,5-diphenyltetrazolium bromide (MTT) reagent (5 mg/ml) was added to each well. After a 3-h incubation at 37°C, 200 μl of DMSO was added to dissolve the formazan crystals, and the absorbance was then measured at 490 nm using a Digiscan Microplate Reader [4]. Wells without cells were used as blanks and were subtracted as background from each sample. Results were expressed as a percentage of control.

### Identification and quantification of apoptotic cells

Treated cells were stained for 10 min with 1 μM Hoechst 33342 to assess nuclear morphology, and with 1 μM calcein AM and 2 μM propidium iodide (PI) to evaluate membrane integrity. About 100 cells were examined in each field at 200× magnification, and at least five random fields were chosen [4].

### Statistical analysis

MTT and cell apoptosis assays were performed in triplicate in three separate experiments. Statistical analysis was performed using SPSS17.0. Results are presented as means ± SD. Data analysis was performed using one-way ANOVA. The level of significant difference was set at *P*<0.05 and *P*<0.01, as indicated.

## Results

### Clinical features of patients

Twenty patients with WD were included in our study. The mean age of disease onset was 17±10 years (range 2–41 years), seven (35%) were males. The 20 patients were the offsprings of 18 couples, and two of the 18 couples (11.1%) were consanguineous. Patients were classified phenotypically into hepatic (n = 11, 55%) and neurological (n = 9, 45%) forms of WD based on initial symptoms or features. The most frequent symptoms or signs in our WD patient population were of hepatic origin, including hepatomegaly (19/20, 95%), splenomegaly (17/20, 85%), jaundice (10/20, 50%), abdominal distension (5/20, 25%), abdominal pain (4/20, 20%), and bleeding (2/20, 10%). Neurological manifestations were observed in 12/20 patients (60%). K-F rings was observed in 19 (95%) patients. Low serum ceruloplasmin (64.7±29.7 mg/dl) and elevated 24 h urinary copper excretion (256.7±80 μg/24 h) were present in all patients. All patients were based on clinical manifestations, laboratory findings (low serum ceruloplasmin, high 24-hour urinary copper excretion), and met the criteria of the Leipzig score [5] (Table 1). The initial treatment regimen of our patients consisted of D-penicillamine and zinc sulfate for the 20 patients, which, either stabilized or improved the course of disease in all patients.

### Mutation analysis

Thirty-six sequence variants, including 16 polymorphisms (7 novel polymorphisms: c.411C>A (p.Ser137Ser), c.2289C>T (p.Phe763Phe), c.2502C>G (p.Val834Val), c.3973C>T (p.Leu1325Leu), c.3999G>T (p.Leu1333Leu), IVS8+26A>G, and IVS8+27G>A), in the *ATP7B* gene were observed by screening 18 probands of our 18 Chinese Han families with WD [6] (Figure 1A). Twenty mutations, including 3 nonsense mutations (p.Ser105X, p.Gln388X and p.Gly837X), one deletion mutation (c.2659delG), one splice mutation (c.1708-1G>C) and 15 missense mutations (p.Val145Phe, p.Thr498Ser, p.Asp765Gly, p.Arg778Leu, p.Pro840Leu, p.Gly869Arg, p.Ala874Pro, p.Thr888Pro, p.Thr935Met, p.Pro992Leu, p.Asp1047Val, p.Ile1148Thr, p.Glu1173Lys, p.Ala1295Val and p.Arg1320Ser) were identified by further analysis of these variants in 100 normal controls (All novel mutations and variants included in the article had been submitted to NCBI, Table S2–4). Among these mutations, Four (p.Val145Phe, p.Gln388X, p.Thr498Ser and p.Gly837X) of them are novel *ATP7B* gene mutations [6]–[8] (Figure 1B). Three homozygous mutations and 15 compound heterozygous mutations were found in probands from 2 consanguinity and 16 non-consanguinity families, respectively. Further analysis of family members showed that homozygous or compound heterozygous mutations in the *ATP7B* gene were the cause of WD in our families. Single heterozygous mutation in one of the two copies of the *ATP7B* gene was insufficient to cause this disorder, consistent with a loss-of-function mechanism of the *ATP7B* mutations.

### Haplotype analysis

Haplotype analysis of different families with the same mutation revealed that clear founder effects for p.Arg778Leu (Families M1279, M1524, M1841, M2621 and M4056), p.Pro992Leu (Families M1031, M1038, M1623, M1841, M1856, M4061, M4062 and M4174), p.Ile1148Thr (Families M1807 and M1856) and p.Ala1295Val (Families M41 and M1407) mutations existed in these families (data not shown).

### *In vitro* effects of *ATP7B* silencing

**Figure 2:**
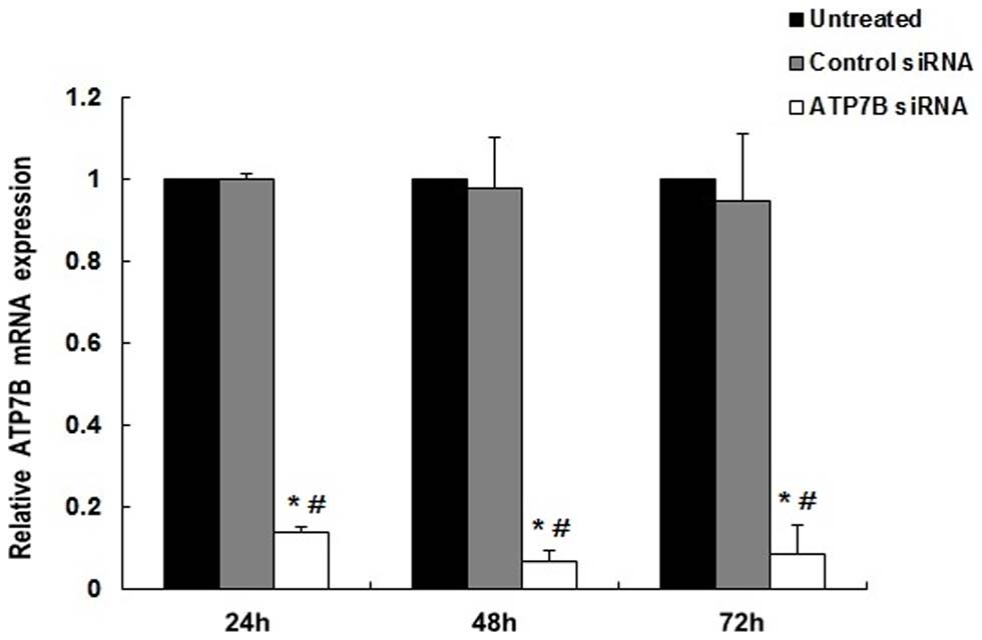
The relative level of *ATP7B* mRNA expression in *ATP7B* siRNA treated cells. Statistical differences between *ATP7B* siRNA transfected groups and AllStars Negative Control siRNA groups are indicated as *(*P*<0.01), or untreated group are indicated as **#** (*P*<0.01).

**Figure 3:**
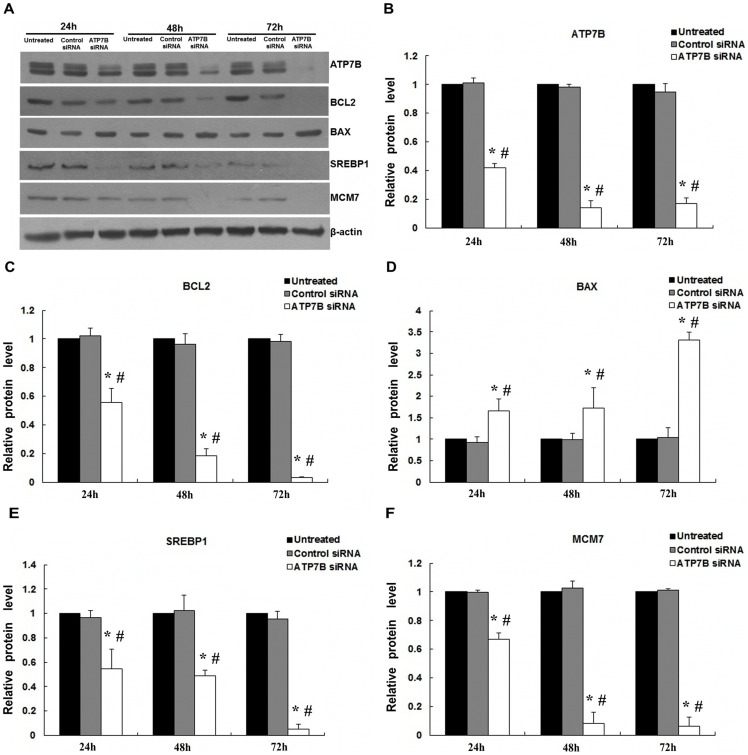
Effects of *ATP7B* siRNA silencing on expressions of *ATP7B*, BCL2, BAX, SREBP1 and MCM7 proteins in HepG2 cell. (A) Western blot analysis of *ATP7B*, BCL2, BAX, SREBP1 and MCM7 in HepG2 cells following treatment with targeted siRNA and AllStars Negative Control siRNA for 24, 48, and 72 h. β-actin was shown below as loading control. Protein levels of (B) *ATP7B*, (C) BCL2, (D) BAX, (E) SREBP1 and (F) MCM7 were quantified by densitometry and expression is shown as arbitrary units. Statistical differences for expression of the proteins between *ATP7B* siRNA transfected groups and AllStars Negative Control siRNA groups are indicated as *(*P*<0.05), or *ATP7B* siRNA untreated group are indicated as **#** (*P*<0.05).

**Figure 4:**
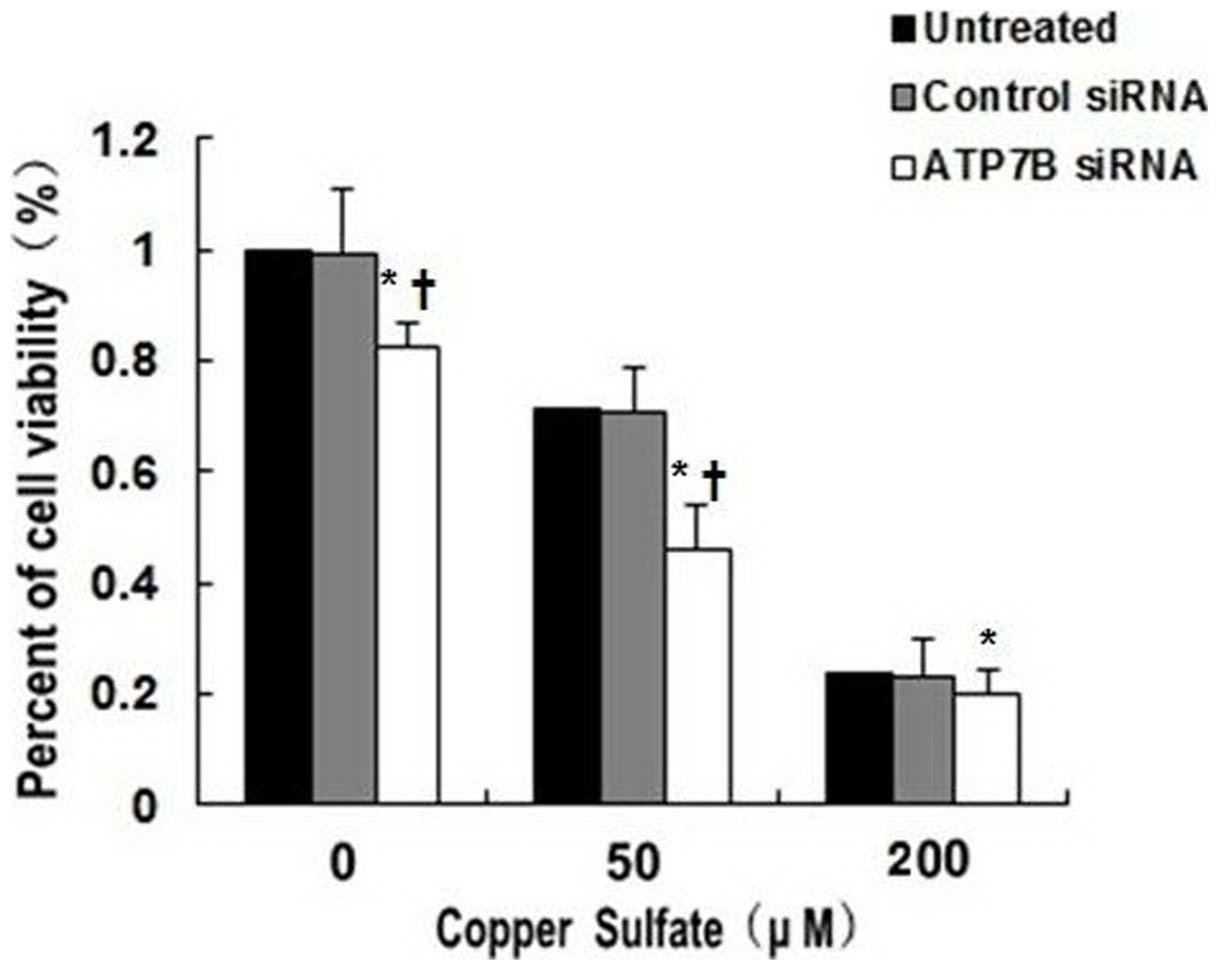
Cell viability of *ATP7B* siRNA treatment to HepG2 cells. *P<0.05 as compared with the AllStars Negative Control siRNA treated cells, †*P*<0.05 as compared within the same concentration of copper sulfate group.

**Figure 5:**
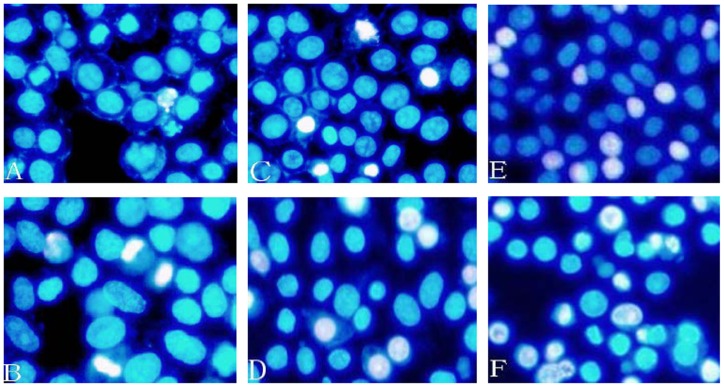
Analysis of apoptosis in HepG2 cells. HepG2 cells were stained with 1 μM of Hoechst 33342. Cells were treated with siRNA for 24 h and followed by copper sulfate treatment for 24 h. (A) Treated with 5 nM AllStars Negative Control siRNA. (B) Treated with 5 nM *ATP7B* siRNA. (C) Treated with 5 nM AllStars Negative Control siRNA+ 50 μM copper sulfate_._ (D) Treated with 5 nM *ATP7B* siRNA+ 50 μM copper sulfate. (E) Treated with 5 nM AllStars Negative Control siRNA + 200 μM copper sulfate. (F) Treated with 5 nM *ATP7B* siRNA+ 200 μM copper sulfate.

Compared to AllStars Negative Control of siRNA treated cells, transfection of HepG2 cells with 5 nM *ATP7B* siRNA resulted in decreased mRNA expression by 86.3%, 93.1% and 90.8% (All *P*<0.01; Figure 2) and decreased protein levels by 58.5%, 85.5% and 82.1% at 24, 48 and 72 hours, respectively (All *P*<0.01; Figure 3A; B). There was no significant difference for mRNA or protein expression between AllStars Negative control siRNA treated cells and untreated cells at 24, 48 and 72 hours (All *P* >0.05). When incubated with 5 nM *ATP7B* siRNA for 48 hours, the cell viability decreased by 17.2% and 16.6% (as compared with untreated cells and AllStars Negative Control siRNA treated cells, respectively) by MTT analysis (*P*<0.05; Figure 4). Using the same incubation method, about 5.6% cells underwent apoptosis (Figure 5).

To examine the combined effects of *ATP7B* deficiency and copper, HepG2 cells were treated with 5 nM *ATP7B* siRNA for 48 hours and then exposed to 50 and 200 μM of copper sulfate for 24 hours. Exposure to 50 μM copper sulfate further reduced the cell viability by 53.3%, as compared with AllStars Negative Control siRNA treated cells alone (*P*<0.05), and by 44.0% as compared with *ATP7B* siRNA treatment alone (*P*<0.05). Cell viability decreased more obviously when *ATP7B*-deficient cells were exposed to higher concentration of copper sulfate (decreased by 79.8% for 200 μM) as compared with AllStars Negative Control siRNA treated cells alone (*P*<0.05), and by 77.6% as compared with *ATP7B* siRNA treatment alone (*P*<0.05; Figure 4). Apoptosis increased to 17.3% and 24.6%, respectively, when cells were exposed to 50 μM and 200 μM copper sulfate (*P*<0.05; Figure 5). *ATP7B* siRNA (5 nM) treatment also increased copper-induced apoptosis accompanying the higher dose of copper sulfate (Figure 5).

### Effect of *ATP7B* silencing on apoptosis, cell cycle and lipid metabolism

To determine if *ATP7B* siRNA treatment may alter translation of apoptosis-related proteins, the expression of the BCL2 and BAX were measured. Real-time RT-PCR analysis showed that the *BCL2* mRNA expression decreased by 90.8%, 95.4% and 96.1% after HepG2 cells were treated with *ATP7B* siRNA for 24 h, 48 h and 72 h respectively, as compared with AllStars Negative Control siRNA treated cells (All *P*<0.05). Whereas *BAX* expression increased by 5.9-fold, 6.4-fold and 7.2-fold at 24, 48 and 72 hours, respectively comparing to those of the AllStars Negative Control of siRNA treated cells (All *P*<0.05). Consistently, Western blot showed that BCL2 decreased to 54.6%, 19.3% and 3.1%, and BAX increased to1.8-fold, 1.7-fold and 3.2-fold for 24 h, 48 h and 72 h (Figure 3A, C, D). These results indicate that *ATP7B* siRNA silencing induced a decrease in BCL2 expression, and an increase in BAX expression, indicative of apoptosis.

Previous *in vivo* data suggested cell cycle machinery and lipid metabolism were selectively affected by copper overload in *Atp7b^−/−^* mice [9]–[11]. Therefore, to explore possible mechanisms in human, we examined the effects of *ATP7B* siRNA silencing on the expression of cell cycle-related gene(s) and lipid metabolism-related gene(s). The mRNA levels of *SREBP1*, a lipid metabolism-related gene, were significantly decreased by 49.7%, 59.9% and 80.0% in the *ATP7B* siRNA silenced cells at 24, 48 and 72 hours, respectively, compared with those of AllStars Negative Control siRNA treated cells (*P*<0.05). Consistently, the protein levels were decreased by 42.7%, 52.2% and 94.9%, respectively. The mRNA levels of the *MCM7*, a cell cycle-related gene, was significantly decreased by 89.2%, 95.1% and 95.5%, and the MCM7 protein levels were significant decreased by 32.8%, 92.4% and 93.6% at 24, 48 and 72 hours, respectively (*P*<0.05; Figure 3A, E, F).

## Discussion

Wilson's disease, a genetic disorder of copper metabolism, is caused by mutations in the *ATP7B* gene. The disease has significant phenotypic diversity, and occurs widely in all ethnic populations [12]. The phenotype of WD usually manifests as a range or a spectrum, and clinical presentations include symptomatic live disease, neuropsychiatric disorders, and other features (hematologic, renal, etc). Affected patients in our families began to have classical symptoms of WD with hepatic or neurological features, and K-F ring, from 2–41 years of age. Our cases also shared clinical features similar to other *ATP7B* cases, including beneficial response to D-penicilliamine [13]. The age of onset is similar within sibships in our study (Family M41 and Family M1279), which may be attributed to the similar genetic background [14]. All of patients had hepatic diseases, whereas only 60% (12/20) of patients had neurological presentations as the early manifestations. Although the hepatic presentation was assumed to mean more severely deranged *ATP7B* function, modifier genes, such as the 5,10-methylenetetrahydrofolate reductase gene, environmental factors, such as nutritional copper intake, infectious disease, drug and toxin, and other epigenetic factors must be at play in the overall phenotypic expression of WD in individuals [15], [16]. We found three homozygous *ATP7B* mutations and 15 compound heterozygous mutations in 2 consanguinity pedigrees and 16 non-consanguinity pedigrees, respectively. Four of these 20 mutations are novel. Among the novel mutations identified in this study, p.Val145Phe was present in two compound heterozygous patients (sib) and their unaffected mother was in heterozygous status (M1279). p.Thr498Ser was found in a 20-year female WD patient (M1038, II:1) with compound heterozygous mutations (p.Thr498Ser and p.Pro992Leu), and the age at the onset was 12 years with neurological presentation. Compound heterozygous *ATP7B* mutations (p.Gly837X/p.Pro992Leu) were found in an 18-year WD female (M1031, II:1) presented with liver disease at the onset age of 2 years old, the earliest reported onset age. All these four novel mutations were not detectable in 100 normal controls. Some reports suggested that the early onset of severe liver disease occurs with frameshift or nonsense mutations [17]. However, our study does not confirm that, consistent with other reports [18], [19]. Homozygous Pro992Leu mutation, probably involved in poor phosphorylation, was found in a 36-year old female patient, she developed abdominal pain and vomiting at age of 18 years, rapidly progressed to acute liver failure and received urgent liver transplantation. On neurological examination, abnormal involuntary movement, festinating gait, dysmetria and bilateral intention tremor on knee-to-heel and finger-to-nose testing, dysdiadochokinesis, and scanning dysarthria were found. Homozygous Ile1148Thr mutation, expected to impair the function of the WD protein by altering the secondary structure of the ATP loop, was found in a 43-year old female with both hepatic and neurologic manifestations. She developed abdominal pain and jaundice at 41 years of age, and abnormal rigidity, bilateral static tremor and dysarthria were found on neurological examination. Although consanguineous marriage were denied, parents from the same village and the identical haplotype between the parents who are from the same village indicates that the mutation may be inherited from a same ancestor. Homozygous p.Ala1295Val mutation, located in the conserved adenosine triphosphate (ATP) hinge region, was found in a female with hepatic manifestation as early symptoms, confirming the association between homozygous mutation in the ATP hinge region of *ATP7B* and hepatic phenotype [20]. p. Arg778Leu and p.Pro992Leu mutations, was found with high allele frequencies in WD form Asia [1], and a similar high allele frequencies (15% for p. Arg778Leu and 22.5% for p.Pro992Leu) were found in our study. Interestingly, haplotype analysis suggested that the different pedigrees existed founder effects for p.Arg778Leu, p.Pro992Leu, p.Ile1148Thr and p.Ala1295Val mutations, suggesting these mutations may be not located on a hot mutation site but due to founder effects.

Several studies suggest that a single *ATP7B* mutation may cause WD [1]. In the few patients in whom only a heterozygous *ATP7B* mutation was detected. It is possible that a second mutation escaped identification by the methods employed or that some mutations in heterozygous forms are sufficient to cause disease [1], [4]. However, in our study, none of the heterozygous carriers were affected, consistent with loss-of-function as the underlying molecular mechanism of *ATP7B* mutations in WD. Therefore, caution should be exercised in the interpretation of heterozygous forms in WD individuals.

Our study indicates that *ATP7B* gene silencing is able to effectively inhibit endogenous expression of *ATP7B* mRNA and protein. *ATP7B* deficiency may reduce cell viability by inducing apoptosis in HepG2 cells. The *ATP7B* deficiency, coupled with exposure to copper, may further aggravate apoptosis in a dose-dependent manner for copper. The increased BAX expression and decreased BCL2 expression in HepG2 cells treated with *ATP7B* siRNA correlates with reduced cell viability and apoptosis.

Our discovery of the reduced expression of SREBP1 and MCM7 mRNA and protein by *ATP7B* silencing in HepG2 cells provides evidence that the deficiency of the *ATP7B* gene is involved in the initiation of the lipid metabolism and cell cycle pathway, although the precise mechanism is unknown. SREBP1 is a fundamental regulator of fatty acid desaturation, elongation and phospholipid biosynthesis. Our results of decreased expression of SREBP1 mRNA and protein by the *ATP7B* inhibition is consistent with the findings in a recent publication of 45 WD patients with the lower levels of total cholesterol and LDL cholesterol compared with the control group [21], and reduced SREBP1 expression in liver of *Atp7b* knockout mice [22].

MCM7 is involved in replicative licensing and synthesis of DNA [23]. In contrast to our result, MCM7 was previously identified as an overexpressed gene in the liver of *Atp7b* knockout mouse in cDNA microarray studies [22]. However, our findings of deceased MCM7 expression in *ATP7B* deficiency cells is consistent with previous observation of overexpression of MCM7 in cancers [24]–[26], which is the opposite extreme for apoptosis.

The detrimental effects of *ATP7B* silencing on hepatocytes may be attributed to its role in promoting reactive oxygen species generation via Cu^2+^ accumulation induced oxidation of biomolecules such as lipids, proteins, and nucleic acids [27], [28].

In summary, 4 novel mutations were identified in our study and apoptosis may result from *ATP7B* deficiency-induced imbalance in cell cycle and lipid metabolism pathway. The results add data to the spectrum of the mutations in the *ATP7B* gene in Chinese Han population.

## Supporting Information

Table S1 **List of real-time RT-PCR primers.**
(DOC)

Table S2 **Polymorphisms detected in Chinese Wilson disease chromosomes.**
(DOC)

Table S3 **Mutations detected in Chinese Wilson disease chromosomes.**
(DOC)

Table S4 **GenBank accession numbers.**
(DOC)
