# Supplementary material for: Novel ATPase Cu2+ Transporting Beta Polypeptide Mutations in Chinese Families with Wilson's Disease
Source: PLoS One. 2013 Jul 2;8(7):e66526. doi: 10.1371/journal.pone.0066526 (PMC3699604; doi:10.1371/journal.pone.0066526)
Supplement: Table S1 — List of real-time RT-PCR primers. (DOC) [file pone.0066526.s001.doc]

**Table S1.** List of real-time RT-PCR primers.

| Gene | Primer Sequence |
| --- | --- |
| *ATP7B* | 5’-GGATTTGAGGCTTCAGTCGT-3’  5’-GAGCCACTTCCTGCACAGAT-3’ |
| *MCM7* | 5’- GCCAAGTCTCAGCTCCTGTC-3’  5’-CTTGGCAATGGAGATGGTCT-3’ |
| *SREBP1* | 5’- ATGGACGAGCCACCCTTC-3’  5’- CAAATAGGCCAGGGAAGTCA-3’ |
| *BCL2* | 5’- ATGTGTGTGGAGAGCGTCAA-3’  5’- TCACTTGTGGCCCAGATAGG-3’ |
| *BAX* | 5’- GTGGCAGCTGACATGTTTTC-3’  5’- GTCTTGGATCCAGCCCAAC-3’ |
| *GADPH* | 5′- GAAGGTGAAGGTCGGAGTCA-3′  5′- GGCAGAGATGATGACCCTTT-3′ |
