# Supplementary material for: Novel ATPase Cu2+ Transporting Beta Polypeptide Mutations in Chinese Families with Wilson's Disease
Source: PLoS One. 2013 Jul 2;8(7):e66526. doi: 10.1371/journal.pone.0066526 (PMC3699604; doi:10.1371/journal.pone.0066526)
Supplement: Table S2 — Polymorphisms detected in Chinese Wilson disease chromosomes. (DOC) [file pone.0066526.s002.doc]

**Table S2.** Polymorphisms detected in Chinese Wilson disease chromosomes.

| Polymorphism | Exon | Nucleotide change | Domain | References |
| --- | --- | --- | --- | --- |
| Ser137Ser | 2 | c.411C>A | Cu1/Cu2 binding domain | **Novel** |
| Ala406Ser | 2 | c.1216G>T | Cu-4 binding domain | Tanzi et al. 1993 |
| Leu456Val | 3 | c.1366C>G | Cu-4/5 binding domin | Tanzi et al. 1993 |
| Phe763Phe | 8 | c.2289C>T | Tm4 domain | **Novel** |
| Leu770Leu | 8 | c.2310C>G | Tm4 domain | Nanji et al. 1997 |
| Arg832Lys | 10 | c.2495G>A | Tm4/Td domain | Figus et al. 1995 |
| Val834Val | 10 | c.2502C>G | Tm4/Td domain | **Novel** |
| Lys952Arg | 12 | c.2855A>G | TM5/TM6 domain | Tanzi et al. 1993 |
| Val1140Ala | 16 | c.3419C>T | ATP loop domain | Tanzi et al. 1993 |
| Val1297Ile | 18 | c.3889G>A | ATP hinge/TM7 | Loudianos et al.1999 |
| Leu1325Leu | 19 | c.3973C>T | Tm7 domain | **Novel** |
| Leu1333Leu | 19 | c.3999G> T | Tm7 domain | **Novel** |
| Possible regulatory |  |  |  |  |
| IVS8+26A>G |  |  | Intron 8 | **Novel** |
| IVS8+27G>A |  |  | Intron 8 | **Novel** |
| IVS18+6T>C |  |  | Intron 18 | Thomas et al. 1995 |
| IVS19+50G>C |  |  | Intron 19 | Cox et al. 2005 |

Novel polymorphisms were boldfaced.
