# Supplementary material for: Novel ATPase Cu2+ Transporting Beta Polypeptide Mutations in Chinese Families with Wilson's Disease
Source: PLoS One. 2013 Jul 2;8(7):e66526. doi: 10.1371/journal.pone.0066526 (PMC3699604; doi:10.1371/journal.pone.0066526)
Supplement: Table S3 — Mutations detected in Chinese Wilson disease chromosomes. (DOC) [file pone.0066526.s003.doc]

**Table S3.** Mutations detected in Chinese Wilson disease chromosomes.

| Mutation | Exon | Nucleotide change | Gene region | References |
| --- | --- | --- | --- | --- |
| Missense |  |  |  |  |
| Val145Phe | 2 | c.433G>T | Cu1/Cu2 | **novel** |
| Thr498Ser | 3 | c.1492A>T | Cu5 | **novel** |
| Asp765Gly | 8 | c.2294A>G | Tm4 | Figus et al., 1995 |
| Arg778Leu | 8 | c.2333G>T | Tm4 | Thomas et al.,1995 |
| Pro840Leu | 10 | c.2519C>T | Td | Loudianos et al., 1998 |
| Gly869Arg | 11 | c.2605G>A | Td/TM5 | [Shah](http://www.ncbi.nlm.nih.gov/sites/entrez?Db=pubmed&Cmd=Search&Term="Shah AB"%5BAuthor%5D&itool=EntrezSystem2.PEntrez.Pubmed.Pubmed_ResultsPanel.Pubmed_DiscoveryPanel.Pubmed_RVAbstractPlus) et al., 1997 |
| Ala874Pro | 11 | c.2620G>C | bet Td/TM5 | Wang et al.,2011 |
| Thr888Pro | 11 | c.2662A>C | bet Td/TM5 | Mak et al., 2008 |
| Thr935Met | 12 | c.2804C>T | TM5 | Wu et al., 2001 |
| Pro992Leu | 13 | c.2975C>T | TM6/Phosphorylation  Domain | Nanji et al., 1997 |
| Asp1047Val | 14 | c.3140A>T | ATP loop | Mak et al., 2008 |
| Ile1148Thr | 16 | c.3443T>C | ATP loop | Loudianos et al., 1998 |
| Glu1173Lys | 16 | c.3517G>A | ATP loop | Loudianos et al., 1999 |
| Ala1295Val | 18 | c.3884C>T | ATP hinge/Tm7 | Tanzi et al., 1993 |
| Arg1320Ser | 19 | c.3960G>C | ATP hinge/Tm7 | Mak et al., 2008 |
| Nonsense |  |  |  |  |
| Ser105X | 2 | c.314C>A | Cu1 | Genschel et al., 2000 |
| Gln388X | 2 | c.1162C>T | Cu4 | **novel** |
| Gly837X | 10 | c.2509G>T | Tm4/Td | **novel** |
| Deletions |  |  |  |  |
| c.2659delG | 11 | c.2659delG | Td/TM5 | Yamaguchi et al.,1998 |
| Splice |  |  |  |  |
| c.1708-1G>C | 5 | c.1708-1G>C | Cu6 | Thomas et al., 1995 |

Novel mutations were boldfaced.
