# Supplementary material for: Novel ATPase Cu2+ Transporting Beta Polypeptide Mutations in Chinese Families with Wilson's Disease
Source: PLoS One. 2013 Jul 2;8(7):e66526. doi: 10.1371/journal.pone.0066526 (PMC3699604; doi:10.1371/journal.pone.0066526)
Supplement: Table S4 — GenBank accession numbers. (DOC) [file pone.0066526.s004.doc]

**Table S4.** GenBank accession numbers.

| Variants in the *ATP7B g*ene | GenBank accession number |
| --- | --- |
| c.411C>A | ss418635409 |
| c.2289C>T | ss418635410 |
| c.2502C>G | ss418635411 |
| c.3973C>T | ss418635412 |
| c.3999G>T | ss418635413 |
| IVS8+26A>G | ss418635414 |
| IVS8+27G>A | ss418635415 |
| Val145Phe | ss418642866 |
| Gln388X | ss418642867 |
| Thr498Ser | ss418642868 |
| Gly837X | ss418642869 |
